# Cryoglobulinemic Glomerulonephritis as a Presentation of Atypical Post-Infectious Glomerulonephritis

**DOI:** 10.14740/jocmr2354w

**Published:** 2015-12-03

**Authors:** Christine Boumitri, Fady G. Haddad, Chetana Rondla, Suzanne El-Sayegh, Elie El-Charabaty

**Affiliations:** a Department of Medicine, Staten Island University Hospital, Staten Island, NY, USA; b Department of Nephrology, Staten Island University Hospital, Staten Island, NY, USA

**Keywords:** Post-infectious glomerulonephritis, Cryoglobulinemia, Acute kidney injury

## Abstract

Post-infectious glomerulonephritis (PIGN) usually occurs within few days to weeks following an infection. Clinical presentation is variable, but in general, it is considered a benign entity with good prognosis. It rarely requires kidney biopsy to confirm the diagnosis. We present a case of a 55-year-old, previously healthy, male who presented for worsening shortness of breath, persistent cough, and right-sided pleuritic chest pain. Initial workup revealed a right exudative effusion with empyema. Hospital course was complicated by acute kidney injury requiring renal replacement therapy with a peak creatinine of 10.2 mg/dL from a baseline of 1.18 mg/dL. On kidney biopsy, findings were compatible with a diagnosis of cryoglobulinemic glomerulonephritis or an atypical form of PIGN. While a wide variety of histopathological findings on renal biopsies have been described to complement the usual diffuse proliferative glomerulonephritis pattern, cryoglobulinemic features with negative cryoglobulin have never been reported. Our case is unique not only by having an atypical histological presentation but also by meeting the criteria of atypical PIGN with persistent hypertension and microscopic hematuria.

## Introduction

Post-infectious glomerulonephritis (PIGN) and the association between infection and kidney injury have been described more than a century ago [1]. Over the last few decades, multiple researchers tried to study the association between the two and it is believed that an Fmune-mediated host reaction to the infectious process is behind the glomerular injury [2, 3]. The first described causative agent of PIGN was streptococcus, thus the name post-streptococcal glomerulonephritis (PSGN) [4]. However, with evolution of medical care and with the introduction of antibiotics, PSGN has decreased in incidence in developed countries while other causes of PIGN have risen such as staphylococcal infection, Gram-negative bacterial infection, as well as fungal and parasitic infections [3, 5, 6]. Typically PIGN occurs within few days to weeks post-infection with a clinical presentation ranging from asymptomatic to full-blown nephritic syndrome [4, 7]. Multiple laboratory values can suggest PIGN as the cause of glomerulonephritis and a kidney biopsy will make the definite diagnosis; however, this is rarely needed since it is considered a benign entity with good prognosis [2, 3]. Most patients will recover within weeks; however, progression to end-stage renal disease (ESRD) has been described in a paucity of cases [8-11]. Multiple reports in the past have described atypical pathological presentation of PIGN [5, 12, 13]. Patients with persistent proteinuria and hematuria after resolution of the infection, who progressed to ESRD or those who did not have any evidence of infection prior to the onset of kidney injury, were labeled “atypical” PIGN [12]. We report a case of atypical PIGN where the clinical picture was highly consistent with PIGN; however, the pathology was not.

## Case Report

### Initial presentation

**Figure 1:**
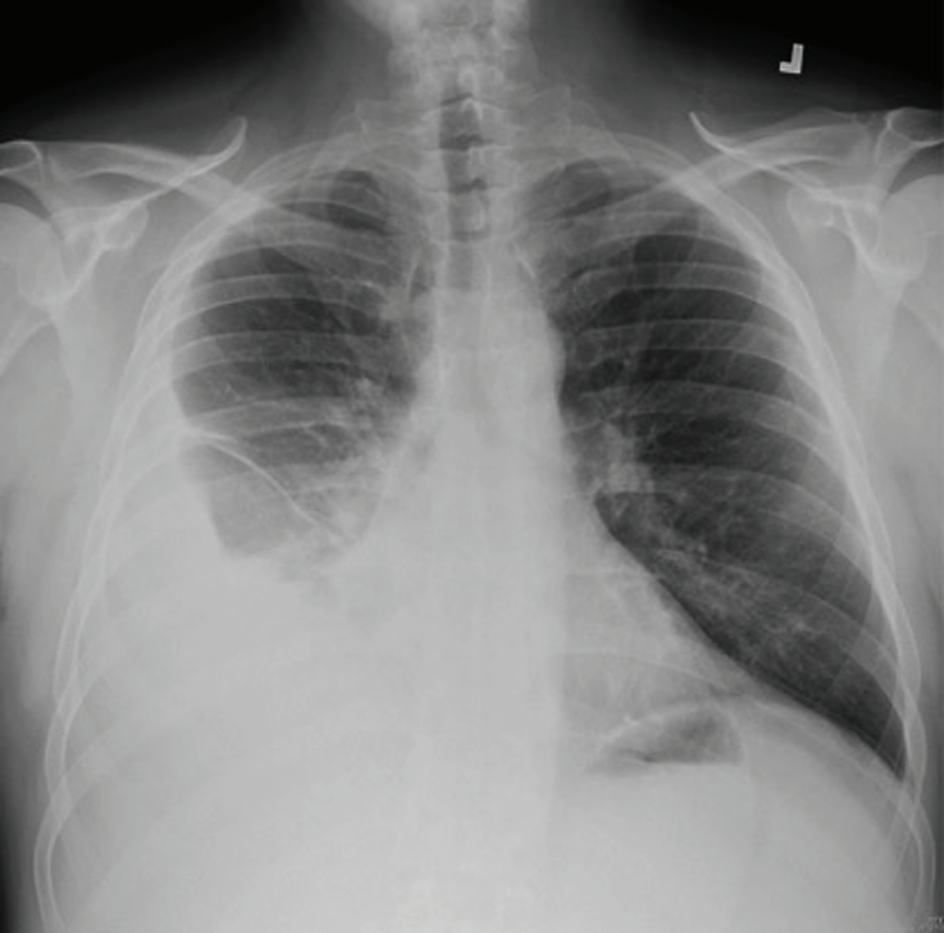
Right pleural effusion with associated basilar atelctasis.

**Figure 2:**
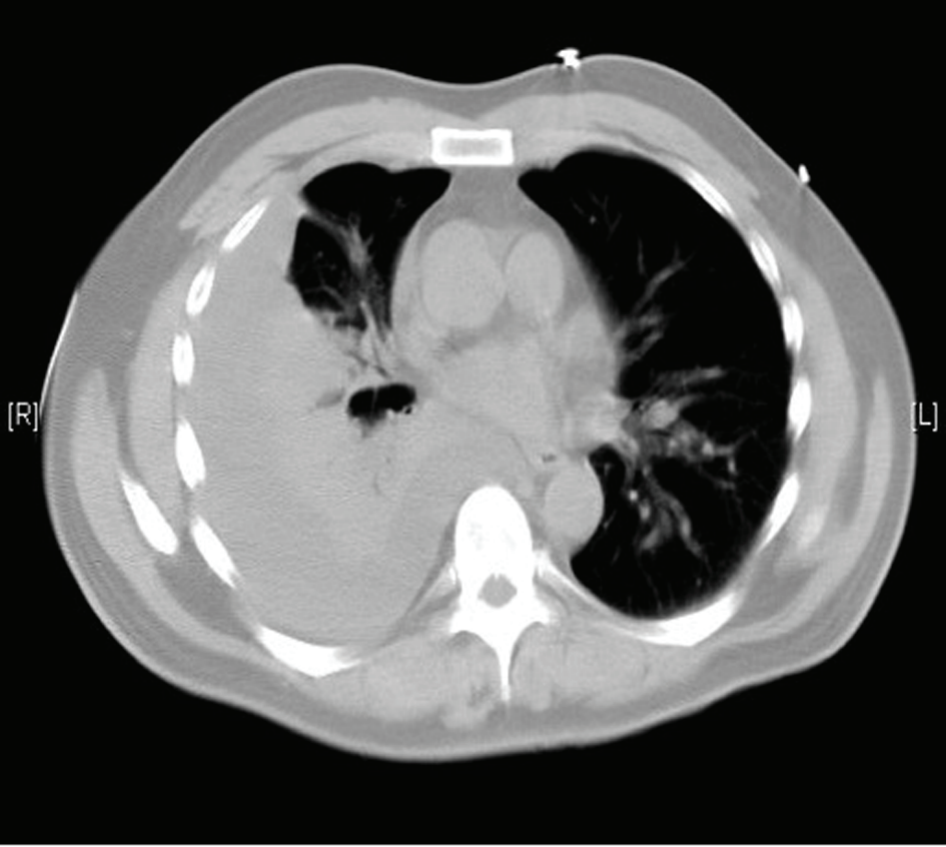
Moderate right pleural effusion.

A 55-year-old previously healthy Caucasian male was referred to us for worsening right-sided pleural effusion. He was seen 1 week prior to presentation in the emergency department (ED) of an outside hospital for shortness of breath and cough, diagnosed with right lower lobe pneumonia with mild pleural effusion and discharged home on oral levofloxacin. One week later, he went to see his primary doctor for worsening symptoms who sent him to the ED. Upon presentation, the patient was complaining of worsening shortness of breath, persistent cough, right-sided pleuritic chest pain, generalized weakness, intermittent low-grade fever and chills. Chest X-ray done in the ED revealed right pleural effusion, which was confirmed by CT scan (Fig. 1, 2).

On admission, vital signs and laboratory studies were normal except for an albumin level of 2.3 g/dL and transaminitis AST/ALT of 113/151 IU/L. Urine analysis revealed trace protein with negative blood. A decision to tap the effusion was made and the patient underwent pleurocentesis revealing exudative effusion with positive empyema. He was started empirically on wide spectrum antibiotics including vancomycin and meropenem and a right chest tube was placed to drain the empyema. On day three of hospitalization, patient’s kidney function started to worsen with a rise in serum creatinine from 1.12 to 1.56 mg/dL with no change in his clinical condition or evidence of hemodynamic instability. A workup for acute kidney injury was initiated.

### Acute kidney injury workup

**Figure 3:**
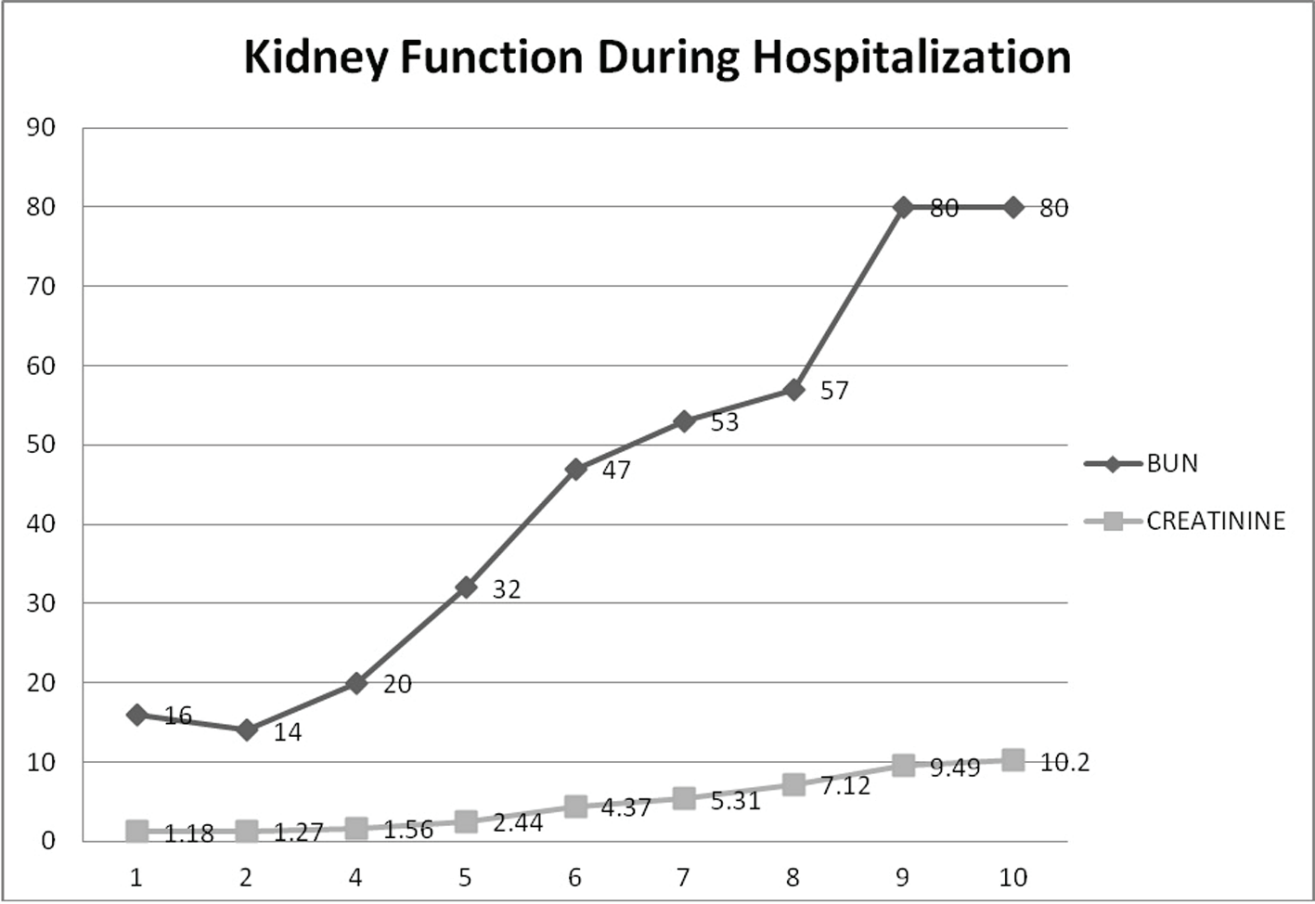
Kidney function during hospitalization. This graph demonstrates the progressive rise in BUN and creatinine during hospitalization until a peak of 10.2 where hemodialysis was started.

A repeat urine analysis showed proteinuria, microscopic hematuria, with no leukocytes or RBC casts. Twenty-four hours urine collection for protein showed a non-nephrotic range proteinuria of 1.6 g. Ultrasound of the kidneys revealed a left kidney size of 11.9 × 6.1 × 5.5 cm and a right kidney size of 11.1 × 6.1 × 5.5 cm. Meanwhile, the patient’s kidney function continued to deteriorate with an increase in serum creatinine to 5.31 on day 7 despite adequate medical management (Fig. 3). All possible offending drugs were stopped and our main differential diagnosis was acute interstitial nephritis (AIN) (initially patient was receiving proton pump inhibitors) vs. acute tubular necrosis (ATN) (patient was taking non-steroidal anti-inflammatory drugs as an outpatient for his pain) vs. glomerulonephritis (PIGN or rapidly progressive glomerulonephritis (RPGN)). Further investigations revealed low complement levels C3 and C4 (35 and 6 mg/dL respectively) with negative hepatitis panel, HIV antibody screen, ANA, ANCA profile, anti-dsDNA, anti-GBM antibodies, cryoglobulin and a normal rheumatoid factor. Blood cultures were all negative. SPEP revealed M spike IgM kappa on immunofixation with faint IgG-lambda and a normal κ/λ ratio.

**Figure 4:**
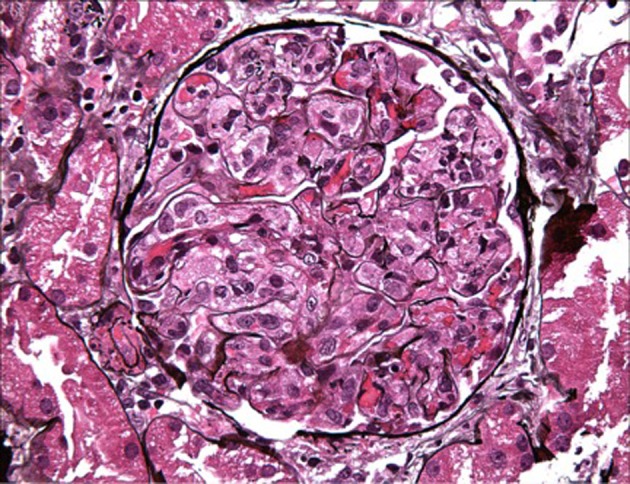
LM - macrophage infiltration.

**Figure 5:**
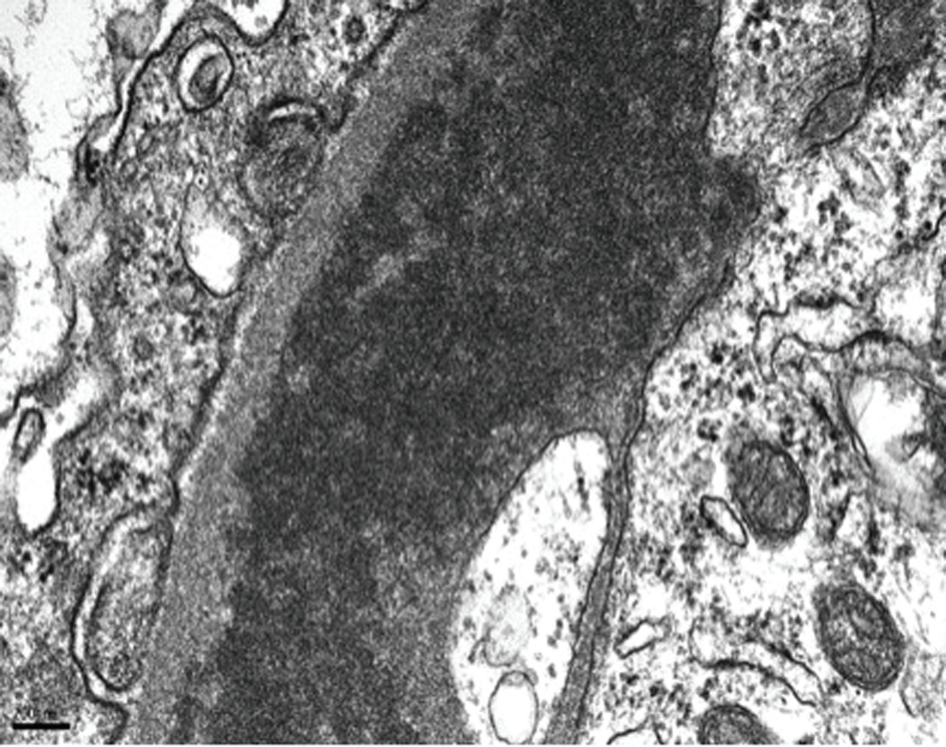
EM - subendothelial deposits.

**Figure 6:**
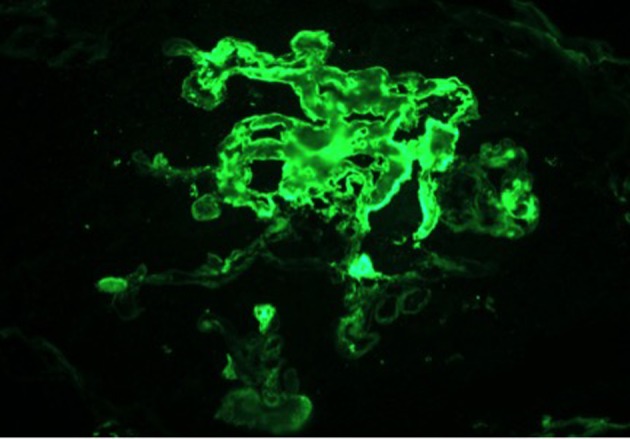
IF - FIBRIN.

**Figure 7:**
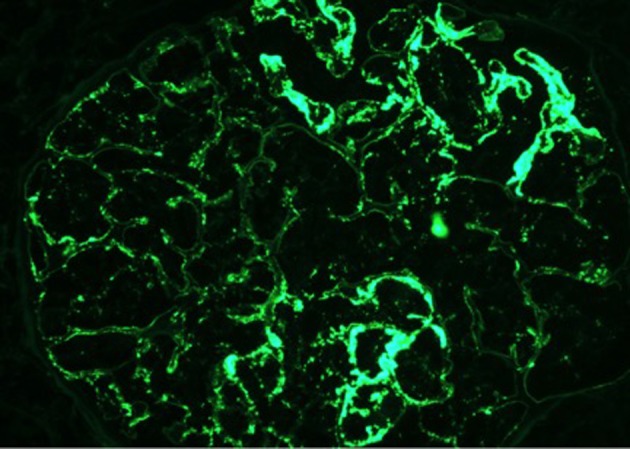
IF - IgG.

On day 7 of hospitalization, patient started to drop his urine output and by the ninth day, his creatinine has risen to 9.49 and he required hemodialysis. A decision to proceed with a core kidney biopsy was made. Results came back as diffuse endocapillary proliferative and exudative glomerulonephritis with infiltrating monocytes/macrophages 3+ and neutrophils 1+ on light microscopy (Fig. 4). Electron microscopy revealed global subendothelial electron dense deposits consisting of mixed cryoglobulin (IgG/IgM) and C3, segmental subepithelial deposits and intracapillary fibrillar fibrin (Fig. 5). Immunofluorescence (IF) revealed global glomerular capillary wall positivity for IgG, trace IgM, 2+ C3, 1-2+ C1, 3+ kappa and trace lambda (Fig. 6, 7). These findings were compatible with a diagnosis of cryoglobulinemic glomerulonephritis or an atypical form of PIGN.

### Follow-up

After three hemodialysis sessions, serum creatinine started to trend down, and patient was making urine again. All cultures came back negative. Repeat complement levels were normal. Patient was discharged after 23 days. Follow-up visit in the office 1 month later showed persistent hypertension, and microscopic hematuria with a serum creatinine of 1.66 mg/dL with trace proteinuria.

## Discussion

PSGN is an immune-mediated glomerular injury resulting from host response to streptococcal infection. In classic cases, it occurs few days to few weeks after streptococcal pharyngitis or impetigo and can affect up to 15% of patients [14]. It usually affects boys with low socioeconomic status; however, its incidence in pediatric population has decreased in developed countries with a parallel increase in elderly patients with risk factors such as diabetes and alcoholism. It usually presents with nephritic syndrome and laboratory findings suggestive of the diagnosis are elevated antistreptolysin O (ASO) and anti-DNAase, low C3 with normal to low C4 levels [2, 3, 15]. Kidney biopsy will typically show global diffuse endocapillary proliferative glomerulonephritis with neutrophilic invasion of the capillary lumen. IF highlights glomerular dominant or codominant C3 granular deposits, while subepithelial hump-shaped deposits are characteristically seen on electron microscopy (EM) [5, 6, 8]. With the antibiotic era, the clinical, epidemiological and pathological spectrum of PIGN has shifted dramatically [2, 3, 6]. Atypical PIGN has been defined as persistent hematuria, proteinuria, and kidney biopsy findings of a PIGN, with or without a preceding infection [12]. Multiple studies have reported an atypical pathological presentation of PIGN on light microscopy, immunofluorescence and electron microscopy.

The classic presentation of diffuse endocapillary proliferative and exudative glomerulonephritis (GN) can be present in 58-72% of cases. An endo-extracapillary GN (with crescents affecting > 25% of glomeruli) has been reported in 9-34% of cases. A focal endocapillary and exudative GN can be present on pathology in 12.8%, while mesangioproliferative GN accounts for 8.1% of cases. The latter might represent a later phase of the disease [16]. Membranoproliferative GN is present in 2.3-8% of cases [5, 6].

While the typical glomerular deposits identified on IF are C3 with or without IgG, C1 was seen in 35.7%, while IgM and IgA were identified in 44% of cases each [6]. On electron microscopy subepithelial deposits were seen in 94% of cases, followed by mesangial deposits in 90.4% and subendothelial deposits in 74.7% of cases [6]. The location of these deposits can be attributed to the stage of renal disease where deposits are mainly subepithelial in the early and acute phase, in the subendothelium and mesangium in the subacute phase, and only in the mesangium in the healing phase [3].

Our case was atypical pathologically by the fact that deposits on electron microscopy were predominantly subendothelial and consisted of mixed cryoglobulin (IgG/IgM) with a strongly positive immunofluorescence for IgG and kappa. These findings were suggestive of cryoglobulinemic glomerulonephritis.

Multiple reports have tried to find an explanation for the inconsistency in the pathologic presentations. One hypothesis is the increased recognition of resolving PIGN at the time of renal biopsy (with atypical PIGN being hypothesized as a resolving phase of typical PIGN) [2].

Also the change in the spectrum of PIGN has been attributed to increase in MRSA infection which is now endemic in hospitals and its association with the atypical IgA-dominant mesangial GN [2]. Nasr et al reported that patients with underlying diabetic glomerulosclerosis had different pathologic features than patients with pure PIGN, with predominance of mesangial and subendothelial deposits, with IgA being the most common immunoglobulin especially with staphylococcal infection [6].

Sehti et al hypothesized that atypical PIGN is the result of defect in the activated alternative complement pathway. It includes mutations in complement-regulating proteins and antibodies to the C3 convertase where the alternative complement pathway remains active even after resolution of the infection. This will result in continuous complement deposits in the kidneys and persistent inflammation and will account for persistent proteinuria and hypertension even after resolution of the infection.

Notably the increased prevalence of non-streptococcal infection such as staphylococcus and Gram-negative bacteria, and the increased occurrence of PIGN in immunosuppressed adults might have influenced the pathologic presentation as well.

While acute PIGN tends to resolve with return to normal kidney function, a progression to persistent renal damage or ESRD has been described [4, 6, 10, 11]. Whether the atypical presentation is associated with worse outcome is still unclear. Wen et al described two different histological patterns of PIGN in their review article; however, no difference in the clinical presentation and outcome between the two groups was observed [2].

Over the last decade, multiple factors have been associated with a worse prognosis, of which we mention alcoholism, diabetes and the presence of diabetic glomerulosclerosis, malnutrition, immunosuppression and malignancy [5, 6, 10, 17].

Also age and serum creatinine at biopsy were inversely correlated with complete remission, and serum creatinine at biopsy was only correlated with ESRD [6, 10].

### Conclusion

We conclude that PIGN features acquired new dimensions with the antibiotic era. Current studies emphasize on the occurrence of PIGN following bacterial, viral and parasitic infections other than streptococcus, especially in developed countries, with adults becoming affected more than children, with immunosuppression (alcoholism, diabetes mellitus, intravenous drug use, acquired immune deficiency syndrome, and malignancy) being a key risk factor. Additional new findings consist of progressive instead of a self-limited clinical course, occult or subclinical infections as inciting events, and a wide variety of histopathological findings on renal biopsies replacing the usual diffuse proliferative glomerulonephritis pattern [2, 3, 6]. Despite all the documented atypical presentations of PIGN, cryoglobulinemic features with negative cryoglobulin have not been reported to our knowledge. Our case is unique not only by having an atypical histological presentation but also by meeting the criteria of atypical PIGN with persistent hypertension and microscopic hematuria. Further investigations about the pathophysiology, the cause and the management of this atypical presentation will be needed and might be the beginning of the discovery of a whole distinct entity of glomerulonephritis.
